# Evaluation of the effect of transcranial direct current stimulation on language impairments in the behavioural variant of frontotemporal dementia

**DOI:** 10.1093/braincomms/fcac050

**Published:** 2022-03-29

**Authors:** Clara Sanches, Fanny Amzallag, Bruno Dubois, Richard Lévy, Dennis Q. Truong, Marom Bikson, Marc Teichmann, Antoni Valero-Cabré

**Affiliations:** 1 Groupe de Dynamiques Cérébrales, Plasticité et Rééducation, FRONTLAB team, Institut du Cerveau et de la Moelle Epinière, CNRS UMR 7225, INSERM 1127, Sorbonne Université, Paris, France; 2 Department of Neurology, National Reference Center for « PPA and rare dementias », Pitié Salpêtrière Hospital, AP-HP, Paris, France; 3 Neural Engineering Laboratory, Department of Biomedical Engineering, The City College of City University of New York, New York, NY, USA; 4 Laboratory for Cerebral Dynamics Plasticity and Rehabilitation, Boston University School of Medicine, Boston, MA, USA; 5 Cognitive Neuroscience and Information Technology Research Program, Open University of Catalonia (UOC), Barcelona, Spain

**Keywords:** frontotemporal dementia, language impairment, transcranial direct current stimulation, non-invasive neuromodulation, neurodegenerative diseases

## Abstract

The behavioural variant of frontotemporal dementia is a neurodegenerative disease characterized by bilateral atrophy of the prefrontal cortex, gradual deterioration of behavioural and executive capacities, a breakdown of language initiation and impaired search mechanisms in the lexicon. To date, only a few studies have analysed the modulation of language deficits in the behavioural variant of frontotemporal dementia patients with transcranial direct current stimulation, yet with inconsistent results. Our goal was to assess the impact on language performance of a single session of transcranial direct current stimulation on patients with the behavioural variant of frontotemporal dementia. Using a sham-controlled double-blind crossover design in a cohort of behavioural frontotemporal dementia patients (*n* = 12), we explored the impact on language performance of a single transcranial direct current stimulation session delivering anodal or cathodal transcranial direct current stimulation, over the left and right dorsolateral prefrontal cortex, compared with sham stimulation. A *Letter fluency* and a *Picture naming* task were performed prior and following transcranial direct current stimulation, to assess modulatory effects on language. Behavioural frontotemporal dementia patients were impaired in all evaluation tasks at baseline compared with healthy controls. Computational finite element method (FEM) models of cortical field distribution corroborated expected impacts of left-anodal and right-cathodal transcranial direct current stimulation over the dorsolateral prefrontal cortex and showed lower radial field strength in case of atrophy. However, none of the two tasks showed statistically significant evidence of language improvement caused by active transcranial direct current stimulation compared with sham. Our findings do not argue in favour of pre-therapeutic effects and suggest that stimulation strategies evaluating the modulatory role of transcranial direct current stimulation in the behavioural variant of frontotemporal dementia must carefully weigh the influence of symptom severity and cortical atrophy affecting prefrontal regions to ensure clinical success.

## Teaser

Sanches *et al*. report no language improvement in frontotemporal dementia after applying transcranial direct current stimulation over dorsolateral prefrontal cortices in a double-blind sham-controlled study. Authors discuss alternative stimulation strategies and patient-customized approaches integrating individual anatomical and functional/clinical features.

## Graphical Abstract

**Graphical Abstract:**
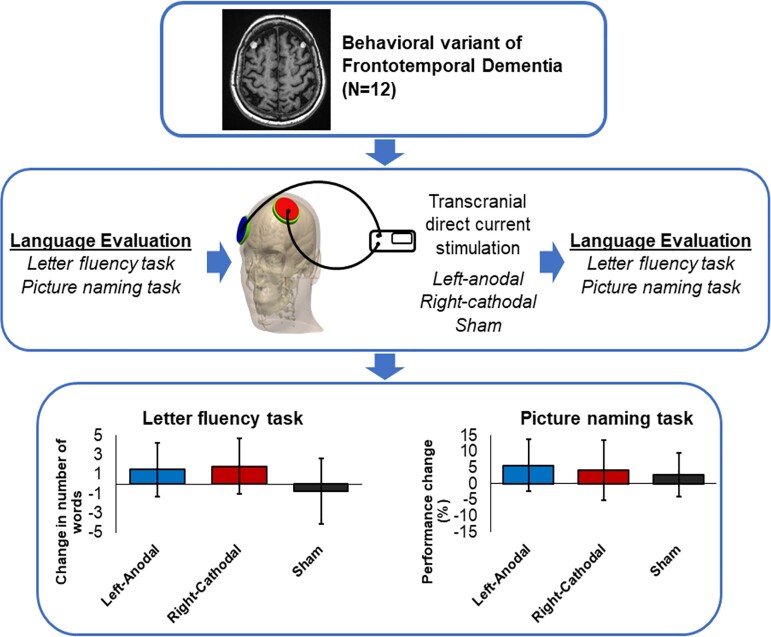

## Introduction

The behavioural variant of frontotemporal dementia (bv-FTD) is an early-onset (<65 years of age) neurodegenerative disease significantly impacting patients’ daily life.^1^ It is characterized by bilateral atrophy of the prefrontal cortex, encompassing dorsolateral, ventromedial and orbitofrontal areas,^2–4^ regions known to subtend executive processes and contribute to the regulation of behaviour.^5,6^

According to the revised diagnosis criteria by Rascovsky *et al*.,^7^ bv-FTD leads to a gradual deterioration of behaviour resulting in apathy, decrease of social convenience, impulsivity and disinhibition. Additionally, executive capacities and language production are severely impaired.^7–10^ Behavioural FTD patients present compromised communication abilities, caused by a progressive breakdown of language initiation/activation and word search mechanisms in the mental lexicon. These disorders are reflected as difficulties in naming and verbal fluency tasks,^11^ which are in part sustained by left dorsolateral prefrontal systems.^12,13^ To date, pharmacological trials in bv-FTD have failed to demonstrate significant benefits, e.g.^14,15^ and no effective treatment for cognitive impairment is currently validated. Speech therapy has shown benefit for language dysfunction, but with no proof-of generalization to untrained items or long-lasting improvements.^16^

With the increase of the elderly population and consequently higher prevalence of neurodegenerative diseases, a crucial need for non-pharmacological neuromodulation therapies is emerging. Non-invasive brain stimulation technologies, such as transcranial magnetic stimulation (TMS) and transcranial direct current stimulation (tDCS) have shown the ability to modulate brain systems and improve cognitive functions such as attention, language, decision making or memory in healthy individuals.^17–19^ Transcranial DCS uses a mild electric field conveyed between two surface electrodes to electrically polarize regions of the cerebral cortex and modulate the membrane potential of exposed neurons, rendering them more or less prone to discharge action potentials and engage in synaptic transmission.^20,21^ Neurodegenerative diseases encompassing large cortical areas such as bv-FTD might prove particularly suited to the spatially broad action of tDCS compared with the higher focality characterizing TMS applications.^22^ Moreover, thanks to its low cost, excellent safety profile and portability, this technique holds promise for increasing activity in the atrophic cortical regions and enhancing cognitive function in neurodegenerative diseases.

Transcranial DCS has already generated beneficial outcomes in several diseases affecting language networks. In post-stroke aphasia, the stimulation of left hemisphere language-related regions boosted the recovery of naming abilities.^23,24^ Moreover, delivered to the dorsolateral prefrontal cortex (DLPFC), tDCS has been shown to induce facilitation of verbal fluency, e.g.^12^ and improvement of lexical access.^25^ In several neurodegenerative diseases, tDCS over the DLPFC has boosted lexical access, such as in Primary Progressive Aphasia,^26,27^ Alzheimer’s disease,^27,28^ Parkinson’s disease^29^ and Progressive Supranuclear Palsy.^13^ However, to date, only a few studies have investigated the effects of tDCS on language impairments in bv-FTD, with inconsistent outcomes.^30,31^ All in all, the low number of patients tested and the marginal level of evidence encourage further investigations.

The present study evaluated the modulatory effects of a single tDCS session applied over the left and right DLPFC on language initiation/activation in bv-FTD patients. We capitalized on interhemispheric inhibition principles,^32^ upon which transcallosal connections mediate rivalrous inhibitory interactions of cortical activity between the two hemispheres. In such framework, we tested and compared across three independent stimulation sessions: (i) anodal stimulation over the left DLPFC aiming to re-boost language processes implemented by language-related prefrontal regions; (ii) cathodal stimulation over the right DLPFC to reduce the inhibition that right hemisphere systems exert on the left dominant language network and (iii) sham tDCS. *Letter fluency* and *Picture naming* tasks were used to evaluate tDCS effects on lexical access and language initiation/activation, whereas a non-verbal executive control task was used to tease apart language-specific effects from an impact on executive processes.

## Materials and methods

### Participants

Twelve bv-FTD patients were recruited at the National Reference Center for ‘Rare Dementias’ of the Pitié-Salpêtrière Hospital in Paris (France). All participants were native French speakers. The diagnosis was established by expert clinicians following international diagnostic criteria for bv-FTD,^7^ including progressive deterioration of behaviour and executive capacities characterized by at least three of the following six criteria: disinhibition, loss of empathy, apathy, perseverative or compulsive behaviours, hyperorality and executive dysfunction, including low verbal fluency. The diagnosis was also supported by neuroimaging (MRI) signs such as the atrophy of prefrontal regions.^7^ None of the patients were under medication interfering with central nervous system activity during their participation in the study.

Exclusion criteria were (i) psychiatric disorders or neurologic diseases other than bv-FTD; (ii) contra-indications to MRI or tDCS, such as the presence of intracranial ferromagnetic devices, scalp or skull lesions or epilepsy and (iii) major depression (Montgomery Asberg Depression Rating Scale > 20),^33^ or major cognitive disorders {[mini-mental state examination (MMSE)] < 15^34^; frontal assessment battery (FAB) < 10^35^}. Fifteen healthy controls, with similar characteristics as patients for handedness, sex, age and years of education (*χ*^2^-test for sex: *P* > 0.05; Mann–Whitney tests for age and years of education: both *P* > 0.05), were used to determine normative performance levels in our evaluation tasks. The study received approval from the local Ethics Committee (Protocol STIMLANG, Ile-de-France I, Paris, France) and written informed consent was obtained from all the participants. Demographic data are summarized in Table 1. Finally, representative MRI images of each of the 12 bv-FTD patients can be found in Supplementary Figure 1.

**Table 1:** Summary of relevant demographic data for bv-FTD patients and healthy control individuals participating in our study (mean ± standard deviation)

	bv-FTD patients	Healthy controls
Number of participants	12	15
Sex (women, men)	4W/7M	8W/7M
Handedness (right/left)	12 R/0 L	15 R/0 L
Age (years)	63.08 ± 11.70	64.13 ± 7.49
Education (years)	14.91 ± 3.73	14.93 ± 2.69
Symptom duration (years)	3.04 ± 2.26	—

### Study design

We applied a double-blind, sham-controlled crossover design in which each bv-FTD patient of our cohort underwent three independent tDCS sessions in 3 separate weeks: anodal tDCS over the left DLPFC, cathodal tDCS over the right DLPFC and sham tDCS over the left DLPFC. Each stimulation session was preceded and immediately followed by a series of computer-implemented evaluation tasks to determine the impact of each stimulation condition tested. The order of the three stimulation sessions was counterbalanced across patients to avoid order biases (six order permutations, two patients for each order). Stimulation sessions were set a week apart to prevent unlikely carry-over effects and ensure independency of interventions. Before the inclusion of the first patient of the study, a computer-generated randomization list was created, and 12 sequentially numbered sealed envelopes, each one containing one of the 12 possible order sets for the three types of stimulation sessions, were stored in a locked research file box. Following the inclusion of each patient, the researcher responsible for delivering the stimulation opened the corresponding envelope and accessed the session order, without revealing such information to any other person involved in the study.

In contrast with TMS protocols, the lack of any accompanying tactile scalp sensations or auditory patterns characterizing tDCS made patients totally unaware of the stimulation condition (anodal, cathodal or sham) delivered on each session. The double-blind character of our design was warranted by asking two different investigators to be in charge either of tDCS application or the application of the evaluation tasks exploring language and executive function.

### Brain stimulation

We followed the same tDCS procedures previously described in different populations of neurodegenerative patients (targeting the right and left anterior temporal lobes in the semantic variant of Primary Progressive Aphasia patients^36^; or the right and left DLPFC in Progressive Supranuclear Palsy patients^13^). In short, electrode placement on the scalp of each patient was guided by means of an MRI-guided stereotaxic frameless neuronavigation system (Brainsight, Rogue Research, Canada), minimizing the Euclidean distance from the skin surface to a well-defined cortical target. Left-anodal and right-cathodal tDCS over the DLPFC regions targeted Montreal Neurological Institute coordinates (*x* = −36, *y* = 32, *z* = 47) and (*x* = 39, *y* = 32, *z* = 45),^13,28^ respectively. A contralateral supraorbital electrode (right and left supraorbital regions, respectively) acted as a return electrode for each montage. The location of active electrodes corresponded to ∼F3 (for left-anodal DLPFC) and ∼F4 (right-cathodal DLPFC) sites of the 10–20 EEG reference system, whereas the contralateral supraorbital return electrode was placed either on AF8 or AF7. Stimulation was delivered through round sponge electrodes [5.65 cm diameter, 25 cm^2^ surface, Neuroelectrics (NE026a) SPONSTIM 25] at an intensity of 1.59 mA (to achieve a current density of 0.06 mA/cm^2^). The current was ramped up for 30 seconds and kept on at 1.59 mA during 20 min before being ramped down for 30 s. Emulating the same transient skin itching sensations characterizing the first and last 10–20 s of active stimulation, the current intensity in the sham tDCS condition was ramped up and down during 30 s at the initial and final phase of the 20-min stimulation blocks.

To ensure safety and measure the level of comfort and tolerance to stimulation, patients were asked to fill a ‘*questionnaire* of *tDCS adverse effects*’^37^ based on a Likert scale assessing patients’ subjective sensations about the most frequent adverse effects reported for tDCS.

### Computational model of electric field distribution

**Figure 1:**
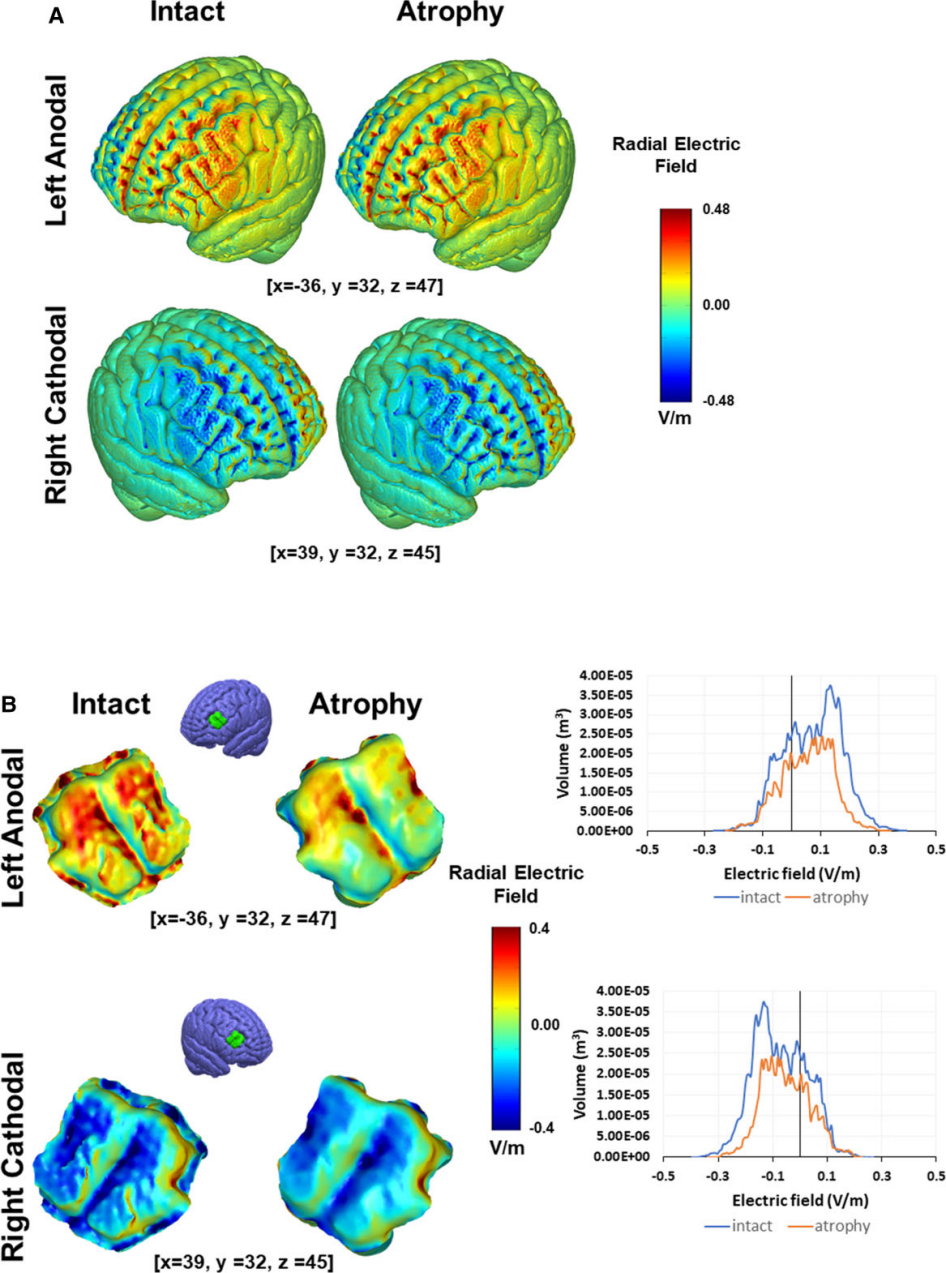
**Biophysical models of tDCS current distribution on targeted cortical areas.** Predicted radial electric field model simulating on a standard head and brain (ICBM-NY) the impact of left-anodal and right-cathodal prefrontal tDCS, delivered with round 25 cm^2^ sponge electrodes (1.59 mA intensity, 0.06 mA/cm^2^ current density) over the DLPFC, with a contralateral supraorbital reference. False colour maps of radial electric field distribution (in V/m; red cortical inward, blue cortical outward) are shown. Specific models shown in the figure compare simulations on a standard intact brain (left) and a brain implementing a degree of cortical atrophy (0.8 mm) in the DLPFC characteristic from bv-FTD patients (right). (**A**) Predicted current flow patterns across the cortical surface. (**B**, left panel) Insets of the DLPFC target for both electrode montages and the intact (left) and atrophied (right) models. (**B**, right panel) Histogram electric field across the DLPFC surface for intact and atrophy predicted models under left-anodal (*top*) and right-cathodal (*bottom*) tDCS.

FEM models were developed on an image-derived standardized head volume [International Consortium for Brain Mapping-New York head (ICBM-NY)]^38^ to determine electric field distributions throughout the head. Electric field was projected onto the surface normal of the cortex to calculate inward (anodal) or outward (cathodal) field. The two tDCS conditions applied in this study were modelled. Further details on tDCS modelling procedures implementing identical parameters (electrode types and montage, current intensity, conductivities, boundary conditions and field equations) can be found in Valero-Cabré *et al*.^13^ The impact of prefrontal cortical atrophy on electric field current density levels impacting the left and right DLPFC was specifically addressed by modelling a cortical thinning (0.8 mm atrophy) of these areas (Fig. 1).

### General cognitive/language assessment

Assessment with standardized tests contributed to the diagnosis of bv-FTD and to the constitution of a relatively homogenous cohort of patients. The general cognitive assessment included the MMSE,^34^ the FAB,^35^ the trail making test A and B,^39^ an evaluation of aphasia severity (Boston Diagnostic Aphasia Evaluation),^40^ a picture naming test (D080^41^) and a verbal fluency test comprising phonemic and category fluency.^42^ Healthy controls were tested with the MMSE and the FAB. Group average test scores for bv-FTD patients and healthy control groups are summarized in Table 2 whereas individual scores for each of the 12 bv-FTD patients of the cohort can be found in Supplementary Table 1.

**Table 2:** General cognitive/language baseline assessments for bv-FTD patients and healthy control individuals participating in our study (mean scores ± standard deviation)

	bv-FTD patients	Healthy controls	Normative thresholds
MMSE	25 ± 4.35	28.33 ± 0.72	≥27
FAB	14 ± 2.02	17.67 ± 0.49	≥16
BDAE—aphasia severity scale	4 ± 0.4	—	>4
Phonemic fluency (P/2 min)	7 ± 4.47	—	≥15
Category fluency (animals/2 min)	13 ± 5.44	—	≥15
DO80	71 ± 7.7	—	>75
TMT A	61 ± 27.29	—	≤40
TMT B	178 ± 90.61	—	≤92

MMSE, mini-mental state examination; FAB, frontal assessment battery; TMT A/B, trail making test version A and version B; BDAE, Boston diagnostic aphasia examination; DO80, picture naming test.

### Evaluation tasks

**Figure 2:**
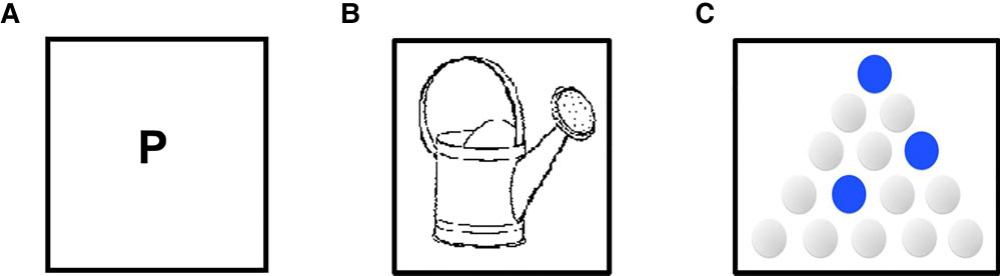
**Computer-based language tasks employed for the evaluation of tDCS effects on language.** Illustration of the three tasks used to evaluate language and executive functions in our study. (**A**) In the *Letter fluency* task patients were requested to provide the maximum number of words beginning with a specific letter in 1 min. (**B**) In the *Picture naming* task patients were asked to name the image in the screen as fast as they could. (**C**) In the *Spatial sequence generation* task patients were required to generate in 1 min the highest number of different sequences made of four dots, without using the blue dots.

Each stimulation session was preceded and followed by language performance assessments aiming to monitor modulatory effects of tDCS following versus preceding stimulation. To limit the confounding of learning effects between the pre- and post-stimulation, each test had two versions matched according to a series of psycholinguistic variables, presented in counterbalanced order across our 12 participants. A *Letter fluency* task was used to assess the initiation/activation of language and lexical access. Participants were asked to generate orally in 1 min as many words as possible beginning with a given letter [‘C’ (version 1) or ‘P’ (version 2)], displayed on the computer screen and provided orally by the examiner (Fig. 2A). Words beginning with ‘C’ or ‘P’ are similar in terms of the number of items and they have a similar cumulative lexical frequency in French (both Fs < 1).^43^

A *Picture naming* task explored the activation of access mechanisms to lexical and semantic representations. The material included the naming of 40 images from two databases of images^44,45^ (Fig. 2B). For both versions of the test, the material was matched for image visual complexity, word lexical frequency^43^ and word and image familiarity. Each image was displayed on the computer screen for a maximum period of 8 s, after which the lack of a response was counted as an incorrect answer.

To control for potential biases linked to the modulation of executive dysfunction, the impact of tDCS on a *Spatial sequence generation* task assessing executive control/attention capacities was also tested. Participants were asked to generate in 1 min the highest number of sequences made of four items (white dots) within a set of 15 items arranged in a triangular configuration (Fig. 2C). They were requested to sequentially select each item, on a touchscreen, with the index finger of their dominant hand and avoid repeating the same sequence or using items appearing in blue (blue dots). The order of the tests preceding or following the tDCS sessions was blocked as follows: (i) *Letter fluency* task, (ii) *Spatial sequence generation* task and (iii) *Picture naming* task.

The two language tasks were programmed with the E-Prime software (Psychology Software Tools, Pittsburgh, PA, USA) and were presented on a laptop computer (HP EliteBook 8770w, USA). The stimuli for the *Spatial sequence generation* task were displayed on a touch-sensitive screen tablet (HP Envy 8 x2). During the task, patients sat in front of the computer, which automatically recorded their responses in the presence of an examiner. The cumulative duration of the three tasks was consistent with the period during which the effects of tDCS are thought to remain active^46^ and significant (∼20 min).

### Statistical analysis

First, performance levels [behavioural scores and reaction times (RTs)] at baseline for the cohort of *n* = 12 bv-FTD patients were compared with those of the *n* = 15 healthy controls, to specify the language impairment in the patient cohort. A non-parametric Wilcoxon signed-rank test was used due to the non-normality of data distribution.

To verify the absence of an across-session learning effect, baseline performances for the three sessions were compared using the non-parametric Friedman test. Regarding stimulation effects, the results obtained in the different tasks (performance scores and RTs) before and immediately after tDCS were compared using the Wilcoxon signed-rank test for each stimulation modality: left-anodal, right-cathodal and sham. The total change in each task (post-stimulation performance minus pre-stimulation performance) was compared across the three stimulation conditions using the Friedman test.

### Data availability statement

Anonymized data, statistical methods and experimental material not entirely published within the article will be shared upon request from any qualified investigator.

## Results

### Computational model of the current density distribution

Both active tDCS stimulation strategies (left-anodal and right-cathodal) were predicted to differentially modulate activity in the lateral and rostral aspects of the targeted DLPFC. The direction of the current flow also indicated opposite modulatory effects.^46^ The presence of mixed polarities between the two stimulation electrodes suggests that stimulation polarity (anodal versus cathodal) depended on the orientation of the electric field relative to the cortical surface. A model simulating atrophy of the left and right DLPFC revealed a decrease in radial electric field strength compared with intact brain models (Fig. 1).

### Evaluation tasks at baseline: patients versus healthy controls

**Figure 3:**
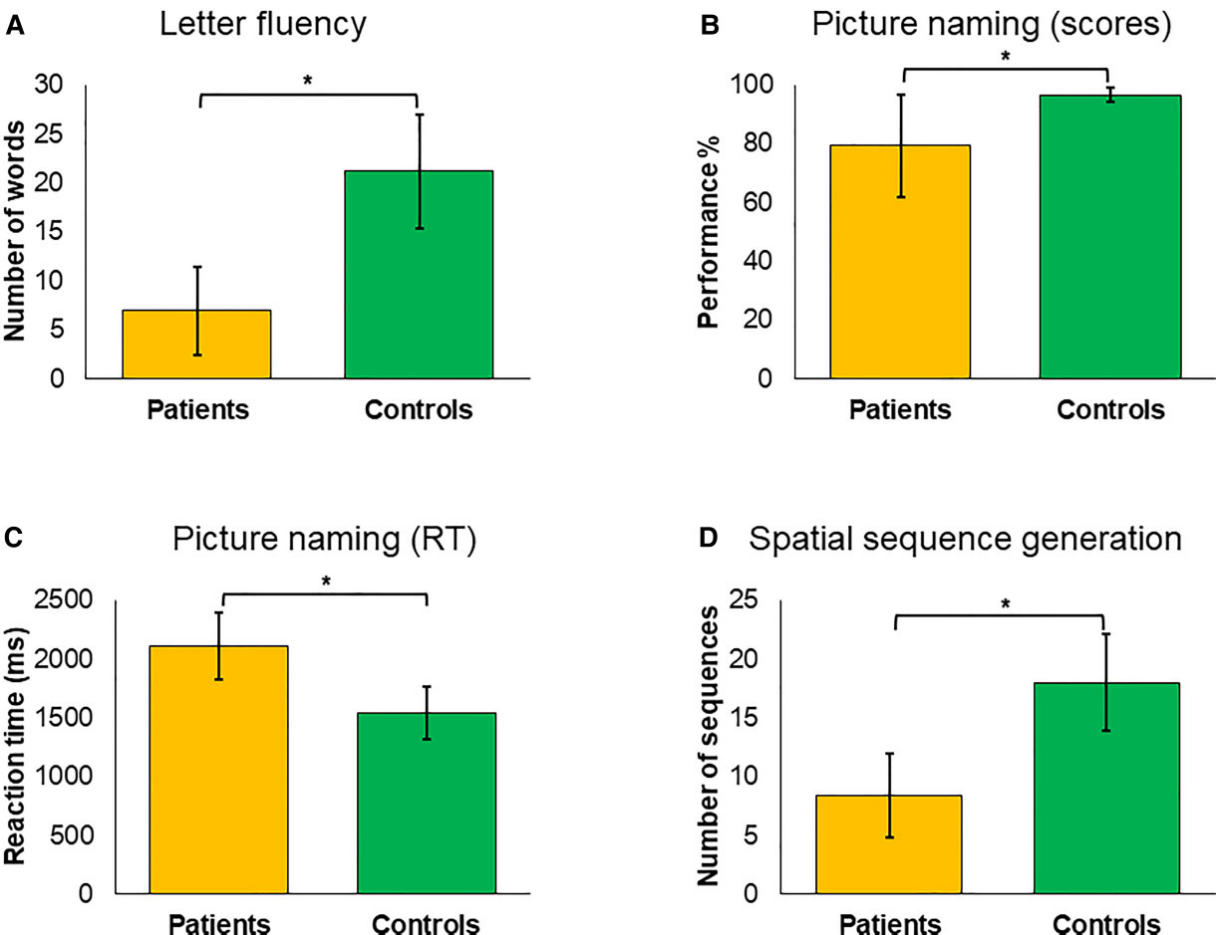
**Baseline language performance in bv-FTD patients versus healthy controls.** Pre-stimulation baseline performance of bv-FTD patients and healthy controls in the three tasks employed in our study to assess the impact of the disease on language and executive function: (**A**) *letter fluency* task (number of words) (*Z* = −6.415, *P* = 7.03e−11), (**B**) *picture naming* task (accuracy scores) (*Z* = −5.283, *P* = 6.32e−8), (**C**) *picture naming* task (RTs) (*Z* = −6.077, *P* = 6.09e−10) and (**D**) *spatial sequence generation* task (number of sequences) (*Z* = −6.113, *P* = 4.86e−10). Notice that patients showed poorer performance than healthy controls in all three tasks. All values are presented as mean ± standard deviation of the mean (standard deviation bars). Non-parametric Wilcoxon signed-rank test served to compare performance between bv-FTD patients (*N* = 12) and healthy controls (*N* = 15).

A comparison of scores and RTs for the 15 healthy controls and the 12 bv-FTD patients showed that the latter performed significantly poorer than the former in the three evaluation tasks used to assess the impact of tDCS on the right or left DLPFC (all *P*-values < 0.001) (Fig. 3).

### Impact of tDCS on language performance and executive function

**Figure 4:**
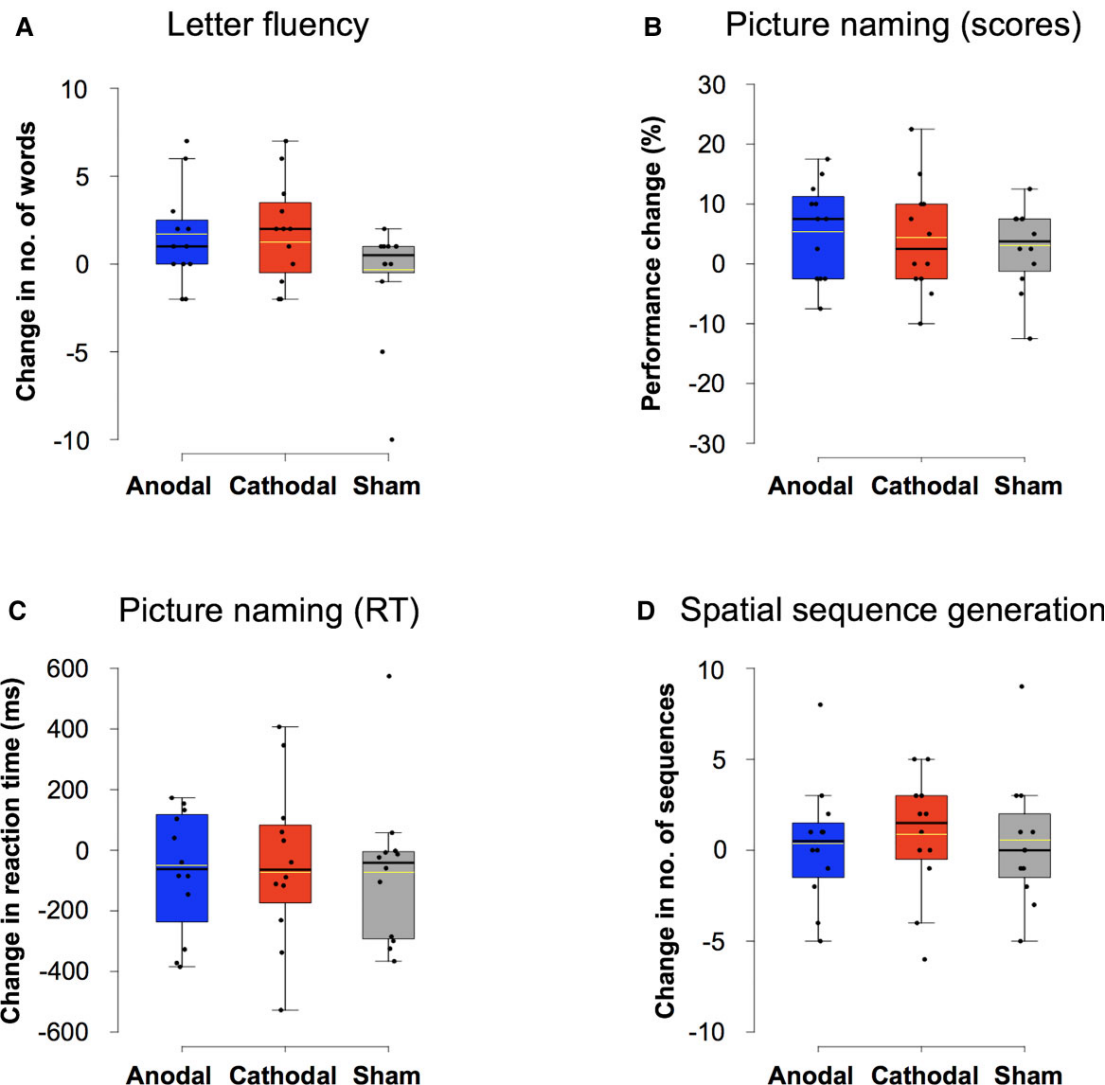
**Language performance modulation by tDCS in bv-FTD patients.** Performance change computed as post versus pre-stimulation outcomes, compared statistically using the Wilcoxon signed-rank test. (**A**) L*etter fluency* task (difference in the absolute number of words produced)—anodal: *Z* = −1.615, *P* = 0.106; cathodal: *Z* = −1.932, *P* = 0.053; Sham: *Z* = −0.245, *P* = 0.807; comparison between the three stimulation conditions—*χ*^2^(2) = 2.909, *P* = 0.234; (**B**) *picture naming* task (difference in accuracy levels)—anodal: *Z* = −1.459, *P* = 0.145; cathodal: *Z* = −1.433, *P* = 0.152; Sham: *Z* = −1.434, *P* = 0.152); comparison between the three stimulation conditions—*χ*^2^(2) = 4.044, *P* = 0.132; (**C**) *picture naming* task (absolute difference of RTs)—anodal: *Z* = −0.706, *P* = 0.480; cathodal: *Z* = −0.706, *P* = 0.480; Sham: *Z* = −1.726, *P* = 0.084; comparison across the three stimulation conditions—*χ*^2^(2) = 0.167, *P* = 0.920 and (**D**) *spatial sequence generation* task (difference in the number of generated sequences)—anodal: *Z* = −0.257, *P* = 0.797; cathodal: *Z* = −0.920, *P* = 0.358; Sham: *Z* = −0.154, *P* = 0.877; comparison between the three stimulation conditions—*χ*^2^(2) = 1.316, *P* = 0.518. Comparison between left-anodal, right-cathodal and sham tDCS was performed using the Friedman test. All *N* = 12. In the box-and-whisker plots presented in the panel, the boundary of the box closest to zero indicates the 25th percentile, a black line within the box labels the median, a yellow line the mean and the boundary of the box farthest from zero indicates the 75th percentile of all values. Whiskers above and below the box indicate maximum and minimum values falling no more or no less than 1.5 times the length of the box. Points above and below the whiskers indicate potential outliers. Note that no significant changes in performance (all *P* > 0.05) were found for any tDCS condition in none of the three tasks evaluated in our study.

A comparison of the number of words produced at baseline by the 12 bv-FTD patients across the three sessions revealed no significant differences [*χ*^2^(2) = 0.706, *P* = 0.703], ruling out across-session learning biases. Nonetheless, the difference between the number of words produced in the *Letter fluency* task immediately before versus after stimulation did not reach statistical significance, regardless of stimulation modality (left-anodal: *Z* = −1.615, *P* = 0.106; right-cathodal: *Z* = −1.932, *P* = 0.053; sham: *Z* = −0.245, *P* = 0.807). Similarly, no statistically significant differences in post- versus pre-stimulation levels were found between the three tDCS conditions [*χ*^2^(2) = 2.909, *P* = 0.234] (Fig. 4A).

Regarding the *Picture naming* task, a comparison of the scores and RT at baseline across the three stimulation sessions revealed no significant differences [scores: *χ*^2^(2) = 3.619, *P* = 0.164; RT: *χ*^2^(2) = 4.167, *P* = 0.125). Likewise, no statistically significant differences were found between pre- and post-stimulation performance for any stimulation modality for scores neither for RT (left-anodal: scores—*Z* = −1.459, *P* = 0.145; RT—*Z* = −0.706, *P* = 0.480; right-cathodal: scores—*Z* = −1.433, *P* = 0.152; RT—*Z* = −0.706, *P* = 0.480; sham: scores—*Z* = −1.434, *P* = 0.152; RT—*Z* = −1.726, *P* = 0.084). Our analyses also failed to reveal statistically significant differences in post-minus pre-tDCS performance across the three stimulation modalities [scores: *χ*^2^(2) = 4.044, *P* = 0.132; RT: *χ*^2^(2) = 0.167, *P* = 0.920] (Fig. 4B and C).

For the *Spatial sequence generation* task, baseline performance was similar for the left-anodal, right-cathodal and the sham tDCS condition [*χ*^2^(2) = 3.244, *P* = 0.197], suggesting, as for our two language tasks, the lack of across-session learning effects and independency of the three stimulation sessions.

**Figure 5:**
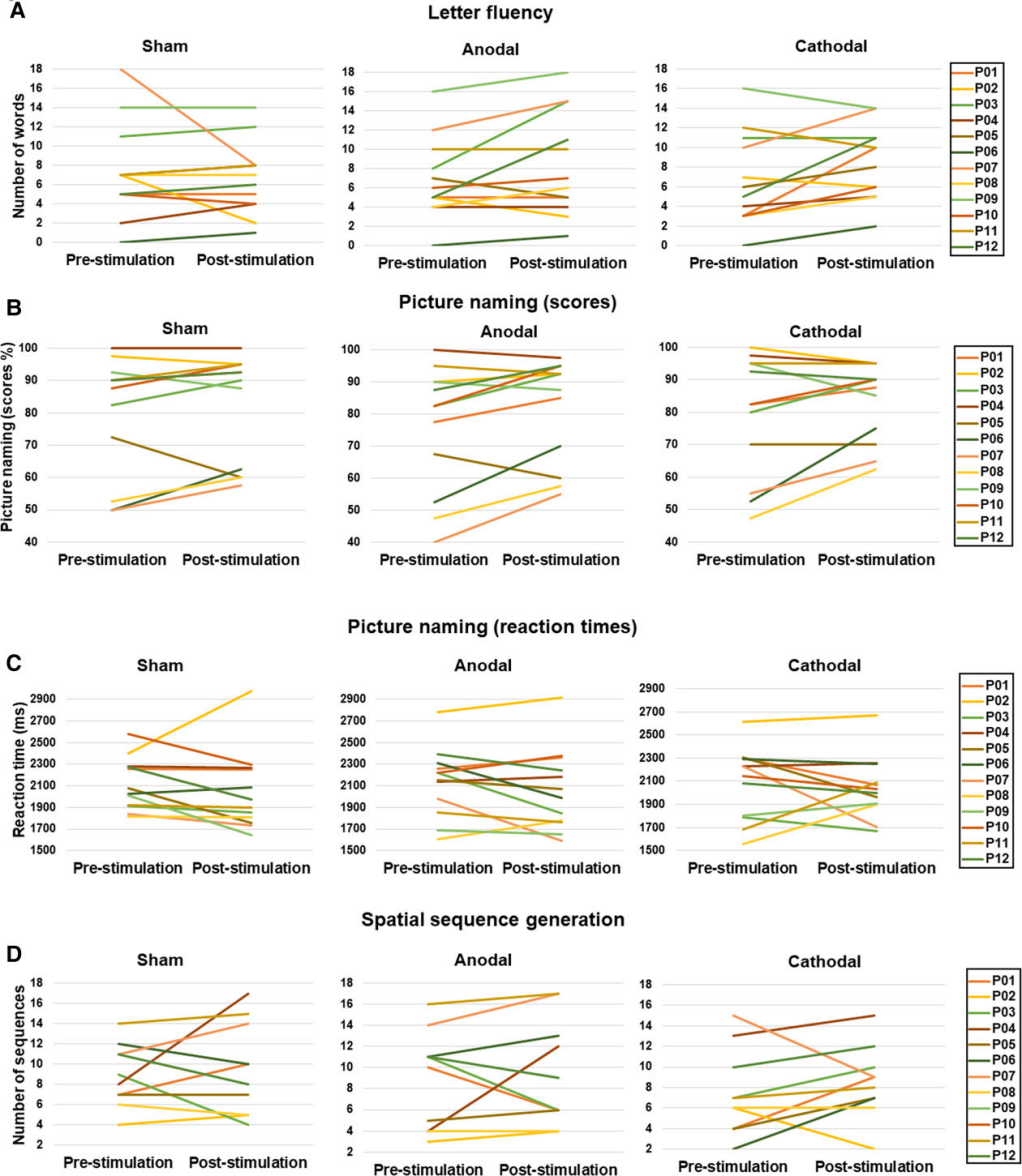
**Individual variability of tDCS effects on language performance.** Performance at baseline (pre-stimulation) and following stimulation (post-stimulation) presented in different colours for each individual patient of our cohort (from P01 to P12) for (**A**) the *letter fluency* task (number of words), (**B**) the *picture naming* task (accuracy), (**C**) the *picture naming* task (RTs) and (**D**) the *spatial sequence generation* task (number of sequences).

Once more, no statistically significant differences were found between pre- versus post-stimulation performance for any of the three stimulation modalities (left-anodal: *Z* = −0.257, *P* = 0.797; right-cathodal: *Z* = −0.920, *P* = 0.358; sham: *Z* = −0.154, *P* = 0.877). No statistically significant differences were found regarding post- versus pre-tDCS differences comparing the three stimulation modalities [*χ*^2^(2) = 1.316, *P* = 0.518] (Fig. 4D). Results at baseline and after stimulation for each individual patient and task are shown in Fig. 5.

## Discussion

Given encouraging results obtained with transcranial stimulation in post-stroke aphasia and in several neurodegenerative diseases affecting language, we here explored the ability of single tDCS sessions to influence language-related networks in bv-FTD. Paralleling prior studies with successful outcomes in early-onset focal neurodegenerative diseases,^13,36^ we applied a double-blind sham-controlled crossover design and compared two stimulation strategies: anodal tDCS delivered over the left DLPFC, aiming to directly stimulate the activity of language-related prefrontal regions and cathodal tDCS over the right DLPFC, intending to suppress interhemispheric inhibitory interactions exerted by this region on left prefrontal regions, hence boosting activity in the latter.

Baseline evaluations yielded significantly lower performance in language tasks for bv-FTD patients compared with healthy controls. This result is not necessarily novel, but it strengthens similar evidence provided by studies reporting verbal fluency and lexical/semantic access deficits in patients with bv-FTD.^8,10,12^ Most important, it reinforces the claim that the left DLPFC is a region involved in the activation of language processes^13,47^ and emphasizes the need to adequately diagnose and attempt to contain language impairments in bv-FTD patients. Nonetheless, contrary to what we predicted, we were unable to reveal statistically significant modulations of language performance in bv-FTD patients following a single session of anodal or cathodal tDCS over the left and the right DLPFC, respectively. The publication of this result is relevant, since the dissemination of non-statistically significant outcomes is essential to moderate publication biases favouring statistically significant beneficial outcomes, hence contributing to a better understanding on ‘if’ and ‘how’ tDCS can be successfully applied to a given neurological condition.

A systematic review of studies using non-invasive transcranial stimulation technologies aimed at improving cognitive impairments in neurodegenerative diseases showed inconsistent outcomes^48^ and emphasized the importance of recruiting well-characterized patient populations embedded in rigorous experimental designs to be able to reliably demonstrate clinical efficacy. In such context, the current lack of success modulating language symptoms which we were able to improve recently using similar tDCS strategies in other neurodegenerative diseases (e.g. Primary Progressive Aphasia and Progressive Supranuclear Palsy^13,36^) calls for a detailed discussion of this discrepancy.

Two explanations seem particularly important to explain the lack of significant language improvements in bv-FTD patients outcomes revealed by our study. First and foremost, the induction of cortical modulatory effects, which following the delivery of a single tDCS session, might have been too weak to supersede a minimal threshold of change in focal activity, hence, to significantly modulate language performance. Supporting this hypothesis, it has been suggested that cumulative effects obtained with periodical multi-day stimulation regimes give rise to the higher magnitude and longer-lasting modulations, linked to the induction of cerebral plasticity phenomena.^49^ Accordingly, significant improvements in verbal fluency have been reported following anodal tDCS over the DLPFC in Parkinson’s disease,^50^ Primary Progressive Aphasia^51,52^ and bv-FTD,^31^ when delivered daily for 10 consecutive stimulation sessions. Similar to our outcomes, highlighting the influence played by the number of accrued stimulation sessions on the significance or the magnitude of the derived clinical outcomes, Huey *et al.*^30^ also failed to find significant improvements in verbal fluency after a single anodal tDCS session over the DLPFC in patients with advanced bv-FTD.

A second factor that has previously been shown to limit tDCS efficiency in well-controlled clinical trials is the baseline severity of the language impairments prior to treatment onset, therefore indirectly also the stage of the disease at which patients suffering a given neurodegenerative condition are proposed with tDCS treatment. To this regard, Pereira *et al*.^29^ reported improvements in phonemic fluency after a single anodal tDCS session over the DLPFC in patients with Parkinson’s disease for whom baseline performance was relatively preserved (17 words/minute) compared with our cohort (7 words/minute). A study evaluating the effects of TMS in a semantic task (word–picture association) in Alzheimer’s disease suggested that patients in the early stages displayed higher improvements than patients at later stages of this condition.^53^ Nonetheless, our limited sample size and the relative homogeneity of baseline scores of our cohort (Fig. 5) did not allow to find clear corroboration in favour of the latter observation. However, in the light of prior studies, we conclude that in order to better individualize interventions, it is paramount to further explore this issue by means of cohorts that could be stratified in different subgroups on the basis of symptom severity.

A related factor that may limit tDCS efficiency is the degree of cortical atrophy affecting the area targeted by tDCS, thus the spared neuronal resources available for modulation. Valero-Cabré *et al*.^13^ have recently used a double-blind sham-controlled design to explore stimulation effects on a cohort of patients with Progressive Supranuclear Palsy and reported improvements in lexical access and language initiation (as measured by a *letter fluency* task) following a single session of anodal tDCS over the left DLPFC. However, Progressive Supranuclear Palsy is characterized by important damage to subcortical structures and relatively mild levels of atrophy in the DLPFC, leaving spared prefrontal neural resources that can be more efficiently boosted by tDCS, than in bv-FTD patients with severely atrophic prefrontal cortices. Strengthening the plausibility of this explanation, a tDCS resting-state functional MRI study in Parkinson’s disease patients characterized by predominant subcortical damage, reported improvements in verbal fluency following anodal stimulation over the left DLPFC along with increased functional connectivity between frontal and inferior parietal regions.^29^ Taken together, these outcomes suggest that anatomical factors such as the severity of cortical atrophy (and the functional impairments derived from such at the excitability, metabolic and neurochemical levels) may influence the efficiency of stimulation regimes.

In the same vein, it has been shown that the volume of the cerebrospinal fluid in the subdural space, gyral depth and the distance between the surface tDCS electrode (anode or cathode) and the cortical target accounted for up to 50% of the spatial variability in electric field strength.^54,55^ Kim *et al*.^56^ modelled individually the current density predicted by tDCS over the left DLPFC in a cohort of healthy adults and reported improvement in a working memory task which correlated with the magnitude of the predicted current density at the cortical target. These findings suggest that inconsistent behavioural outcomes of non-invasive brain stimulation approaches (either tDCS or TMS) might be importantly influenced by interindividual differences of head and brain features, which might become even more variable in patient populations.^57–59^ On this basis, we hypothesize that the high degree of DLPFC atrophy shown by bv-FTD patients at the time of diagnosis may be one of the main causes that limit the beneficial impact of tDCS. It follows that more effective therapeutic approaches, based on a different rationale for the choice of electrode montages, should identify and target relatively spared cortical areas within language networks involved in lexical/semantic access and verbal fluency, rather than focusing on often highly damaged DLPFC areas with scarce viable resources to modulate.

Indeed, language-processing related cortical sites in prefrontal, parietal and temporal regions revealed by neuroimaging studies which might stay undamaged on a given disease could provide alternate targets for neuromodulation.^60,61^ More specifically, in bv-FTD, the temporal–parietal junction and the anterior temporal cortex, involved, respectively, in lexical^62^ and semantic representations,^63^ remain relatively spared, hence should be alternatively considered as more viable targets. Emphasizing the importance to combine adequate cortical targets and suitable stimulation strategies in order to optimally influence cognitive networks, a published study used bilateral anodal stimulation over fronto-parietal regions in bv-FTD patients, which improved scores of the neuropsychiatric inventory and visual RTs.^64^

The use of well-designed double-blind sham-controlled pre-therapeutic studies is paramount to obtain proof-of-concept proving beneficial effects of transcranial stimulation in neurological diseases and, on such basis, justify more costly, larger-scale clinical trials in search of longer-lasting therapeutic outcomes. As successfully achieved for other neurodegenerative diseases,^13,36^ we here aimed to achieve brief lasting improvements of language impairments in bv-FTD patients with a single tDCS session targeting the DLPFC. Our study failed to provide such proof-of-concept. However, together with prior investigations, our results highlight the need to factor in the magnitude of language impairments at baseline and consider the degree of cortical atrophy impacting cortical target regions; therefore, to identify in larger bv-FTD populations the optimal window of severity at which patients might be more responsive to stimulation, and consider the application of tDCS to relatively undamaged regions of the language network. Individual patient approaches might call for a selection of candidates to specific tDCS therapeutic protocols according to their individual anatomical parameters and clinical profile of disease severity. On a very similar basis, a ‘personalized precision medicine’ framework using individually profiled therapeutic approaches based on multiple variables (such as symptom severity and degree of atrophy) may gain ground in brain stimulation. In parallel, biophysically inspired computational models of tDCS generated current fields, individualized to each patients’ head and brain structure, may help tailor the electrode montages and stimulation parameters most suited to optimize outcomes.^65,66^

## Supplementary Material

fcac050_Supplementary_Data

## Abbreviations

bv-FTD =: behavioural variant of frontotemporal dementia
DLPFC =: dorsolateral prefrontal cortex
FAB =: frontal assessment battery
ICBM-NY =: International Consortium for Brain Mapping-New York head
MMSE =: mini-mental state examination
RT =: reaction time
tDCS =: transcranial direct current stimulation
TMS =: transcranial magnetic stimulation
