# Supplementary material for: Evaluation of the effect of transcranial direct current stimulation on language impairments in the behavioural variant of frontotemporal dementia
Source: Brain Commun. 2022 Mar 29;4(2):fcac050. doi: 10.1093/braincomms/fcac050 (PMC8963324; doi:10.1093/braincomms/fcac050)
Supplement: fcac050_Supplementary_Data [file fcac050_supplementary_data.pdf]

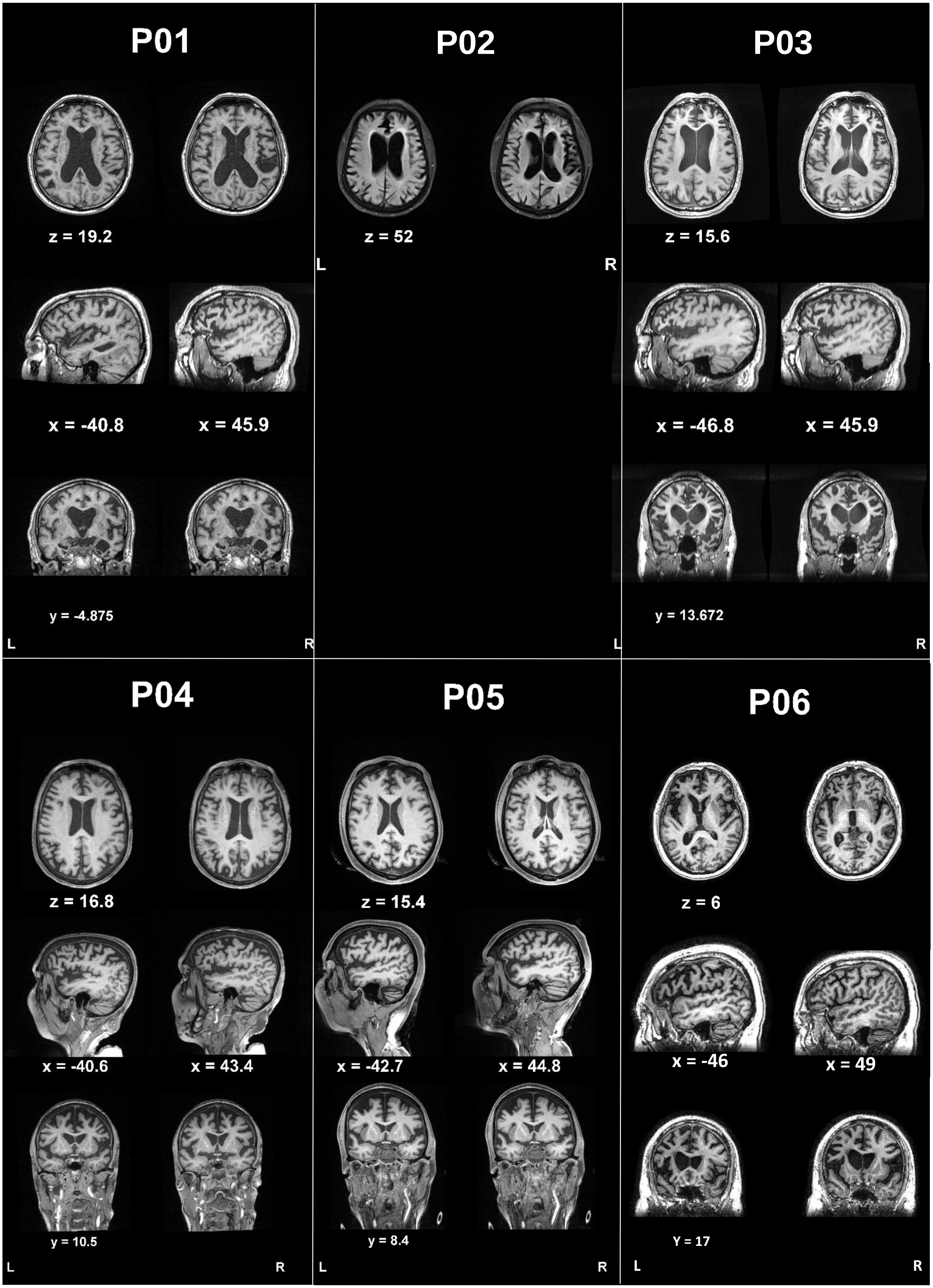
P01
z = 19.2
x = -40.8
x = 45.9
y = -4.875
L
R
P02
z = 52
L
R
P03
z = 15.6
x = -46.8
x = 45.9
y = 13.672
L
R
P04
z = 16.8
x = -40.6
x = 43.4
y = 10.5
L
R
P05
z = 15.4
x = -42.7
x = 44.8
y = 8.4
L
R
P06
z = 6
x = -46
x = 49
Y = 17
L
R

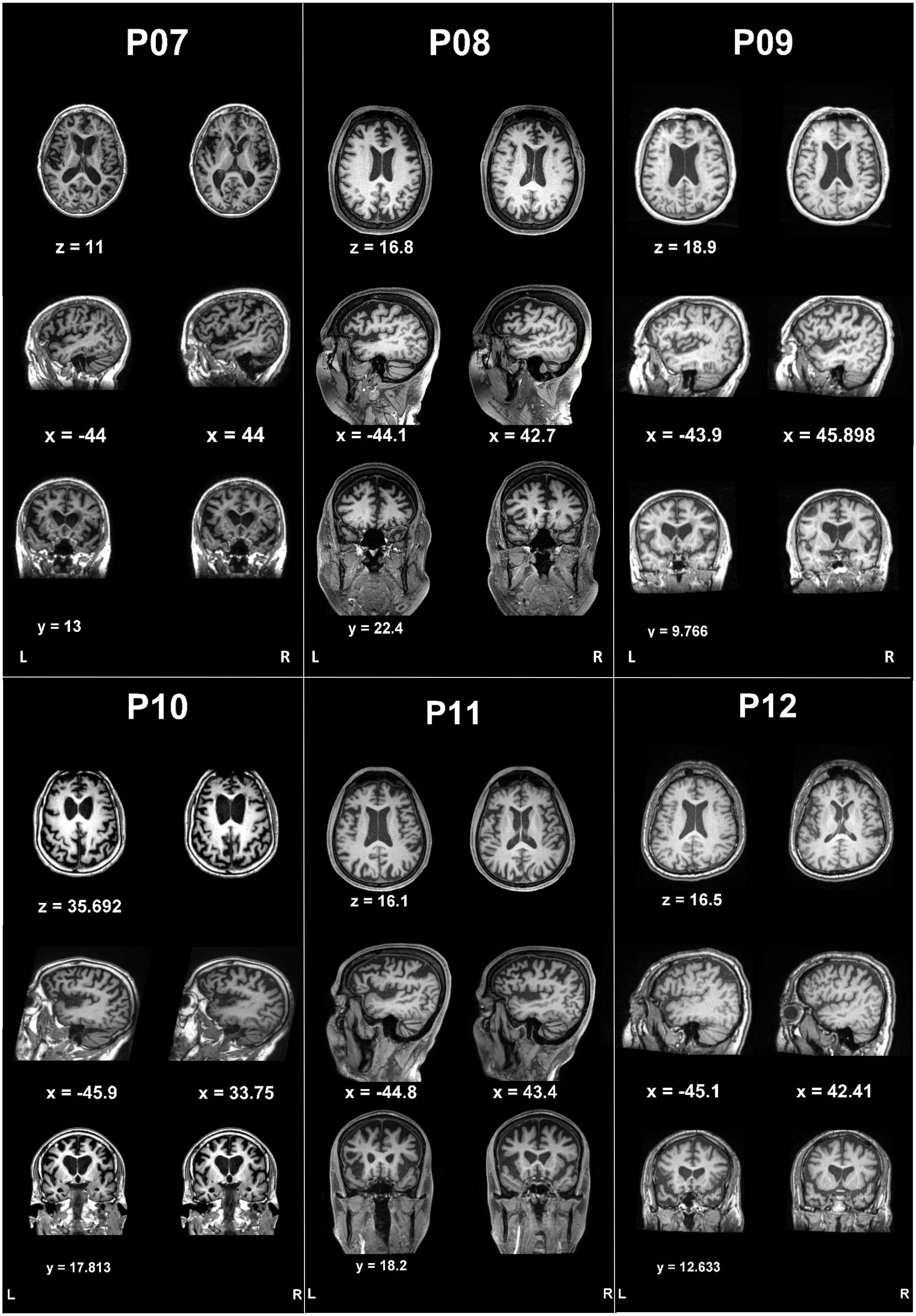

Supplementary Figure 1. Layouts of the structural MRIs of each participant. From left to right and from top to bottom are depicted axial, sagittal and coronal slices of the structural MRIs of each bv-FTD patient included in this study. Images are in the neurological orientation.

Supplementary Table 1. Individual clinical scores in the standard cognitive and language assessment tests employed to characterize our cohort of bv-FTD patients. The twelve patients of the cohort are numbered from top to bottom from P01 to P12.

	**MMSE**	**FAB**	**BDAE - aphasia severity scale**	**Phonemic fluency (P/2min)**	**Category fluency (animals/ 2min)**	**DO80**	**TMT A**	**TMT B**
**P01**	16	10	n/a	3	9	n/a	101	242
**P02**	17	12	4	5	10	68	n/a	n/a
**P03**	28	16	4	14	21	n/a	32	75
**P04**	27	14	4	5	8	68	58	187
**P05**	25	13	4	2	12	68	83	n/a
**P06**	24	12	4	1	8	54	107	n/a
**P07**	27	14	4	12	18	74	38	217
**P08**	29	17	4	13	20	79	46	91
**P09**	28	15	5	8	16	78	38	112
**P10**	24	15	5	5	5	70	64	152
**P11**	23	15	4	7	16	77	42	347
**P12**	29	16	4	n/a	n/a	n/a	n/a	n/a
